# A network structure of manic symptoms

**DOI:** 10.1002/brb3.2010

**Published:** 2021-01-16

**Authors:** Giovanni Briganti, Charles Kornreich, Paul Linkowski

**Affiliations:** ^1^ Unit of Epidemiology, Biostatistics, and Clinical Research Université libre de Bruxelles Brussels Belgium; ^2^ Laboratoire de Psychologie Médicale et Addictologie Université libre de Bruxelles Brussels Belgium

**Keywords:** bipolar disorders, centrality, Granger causality, network analysis

## Abstract

**Objectives:**

The aim of this study is to explore mania as a network of its symptoms, inspired by the network approach to mental disorders.

**Methods:**

Network structures of both cross‐sectional and temporal effects were measured at three time points (admission, middle of hospital stay, and discharge) in a sample of 100 involuntarily committed patients diagnosed with bipolar I disorder with severe manic features and hospitalized in a specialized psychiatric ward.

**Results:**

Elevated mood is the most interconnected symptom in the network on admission, while aggressive behavior and irritability are highly predictive of each other, as well as language‐thought disorder and “content” (the presence of abnormal ideas or delusions). Elevated mood is influenced by many symptoms in the temporal network.

**Conclusions:**

The investigation of manic symptoms with network analysis allows for identifying important symptoms that are better connected to other symptoms at a given moment and over time. The connectivity of the manic symptoms evolves over time. Central symptoms could be considered as targets for clinical intervention when treating severe mania.

## Graphical abstract

In this work, manic symptoms are studied as a network of mutually influencing components in a sample of severe inpatients. Elevated mood is the most interconnected symptom.

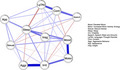

## SIGNIFICANT OUTCOMES

- Elevated mood is the most interconnected symptom in the manic network on admission.
- Aggressive behavior is connected to irritability, and language‐thought disorder to abnormal content.
- The network approach can be useful to monitor the evolution of the connectedness of manic symptoms over time.

## LIMITATIONS

- The assumption of stationarity may be violated in our sample.
- Spearman's rho was used as correlation input instead of Pearson's correlation coefficient.
- Only three time points were measured in this study.

## 1 INTRODUCTION

Investigating the way symptoms of mental disorders arise in a given patient or group of patients has always been a domain of interest in psychiatric research (Kendler et al., 2011). In the past decades, the fields of clinical psychiatry and psychiatric epidemiology have tried to formalize theories around such interactions using models that reflect the complex nature of mental disorders, such as the network theory (Borsboom, 2017). When considering a mental disorder as a network, symptoms are defined as components that cause each other, and the set of interactions that are shared among symptoms determine the mental disorder itself (Borsboom & Cramer, 2013); symptoms are therefore considered as more than observable consequences of the mental disorder, but actively contribute to it. The interactions among symptoms can be computed with a set of statistical techniques called network analysis (Epskamp et al., 2017).

The network approach to mental disorders is helpful when dealing with the important aspect of mental disorders that is heterogeneity (Fried et al., 2016; Fried & Nesse, 2015): Symptoms are not all correlated with each other, some are interconnected in a network more than others and are therefore relatively more important, or “central” to the mental disorder (Briganti & Linkowski, 2019). Network models allow for investigating both contemporaneous (cross‐sectional) and temporal effects of symptoms on each other: the former represent how symptoms interact at a given moment, and the latter show how symptom influence each other over time (Epskamp, 2019). Current diagnostic manuals, such as the DSM‐V (American Psychiatric Association, 2013), do not offer sufficient insight on heterogeneity because of their categorical approach to mental disorders (and therefore that symptoms are interchangeable). Network models, because of their complex nature, can help us identify which are important symptoms in a given disorder, so that they could serve as prime targets for clinical intervention if identified (Blanken et al., 2019). In network analysis, important nodes can be identified based on their quality to predict (or be predicted by) other nodes in the network and therefore their connectivity. Such important nodes are customarily defined as “predictive” or “predictable.” However, identifying central items in cross‐sectional networks is just the first step toward the identification of suitable targets for clinical intervention, since cross‐sectional network analysis cannot, for instance, inform on the direction of causal pathways between symptoms. Identifying the best targets for intervention is all the more important in severe forms of psychiatric disorders, for instance in cases where intervening on specific symptoms can rapidly improve a patient's mental condition.

Acute mania is an example of such a situation where rapidly acting on “central” symptoms can change the course of the severe episode (Vieta & Sanchez‐Moreno, 2008). One could for instance pharmacologically act upon one highly connected symptom to change the state of other symptoms that are connected to it. Network models have been used to investigate bipolar disorders to identify such central symptoms; mood symptoms were found to be the most interconnected in a sample of minimally impaired, depressed and cycling patients (Koenders et al., 2015), as well as in a sample of adolescents with or at risk of bipolar disorder (Weintraub et al., 2020); although the two studies previously investigated bipolar symptoms, their samples were not focused on patients undergoing an acute episode, and only contemporaneous effects were investigated. Predictors of lithium response and relationships between positive and negative effects have also been investigated using network models (Curtiss et al., 2019; Scott et al., 2020).

Inspired by the insight that the network theory of mental disorders can add to mania, we aim to study manic symptoms as a network of mutually influencing elements in a sample of inpatients with acute mania. To investigate a network structure of manic symptoms, it is important to determine which variables to include: Popular psychometric and clinimetric tools have been argued to serve as a good starting point for such a situation (Robinaugh et al., 2019). The Young Mania Rating Scale (YMRS) is a widely used tool to assess the severity of mania and is composed of eleven symptoms (Young et al., 1978). Because the number of symptoms is relatively low compared to other scales, the variables in the YMRS can be a good input for a network model because the estimates are likely to be more stable in a reasonable sample (Epskamp et al., 2017).

Investigating mania as a network can provide interesting new information on our understanding of bipolar disorders: We hereby describe three domains upon which the network approach can shed light.

First, network analysis can retrieve what features of mania are more important compared to others in a network; although it is reasonable to hypothesize that in a network structure of manic symptoms elevated mood and irritability would be highly connected symptoms, because of the nature of bipolar I disorder itself, identifying other potential central features of the manic symptomatology, like increased energy emerged as a central symptom in recent works (Weintraub et al., 2020). Second, network analysis can discern if symptoms connect in a different way in a number of situations (e.g., sex differences, before/after treatment): A different connectivity leads to overall different network structures in the observed sample and can be investigated through network comparison tests (van Borkulo et al., 2016). Third, temporal networks can compute the effects that symptoms have on other symptoms over time (with the help of treatment); this principle is driven by Granger causality in panel data (Granger, 1969); in the case of mania, it is for instance interesting to identify if certain symptoms are less affected than others after treatment (e.g. they are poorly connected and/or reinforce themselves); this may be the case for insight, since it has been identified before as an independent predictor of outcome (Cassidy, 2010).

This work is organized as follows: first, the cross‐sectional network structures of mania at the start, middle, and end of hospitalization are estimated. Second, node predictability (shared variance of a node with surrounding nodes) is studied as a measure of absolute connectivity and therefore importance in the network structures (Haslbeck & Fried, 2017). Third, the differences between the cross‐sectional networks (start, middle, and end of hospitalization) are estimated regarding the strength of connections (van Borkulo et al., 2016). Fourth, a temporal network is estimated from the three time points to investigate the temporal effects among symptoms.

## 2 METHODS

### 2.1 Data sets

#### 2.1.1 Patients

Our data set is composed of 100 patients, hospitalized in the context of an involuntary commitment in a secure psychiatric ward specialized in mood disorders and psychosis in 2019. The data were collected retrospectively. To be included in this study, patients had to be diagnosed before or during the psychiatric expertise preceding the admission (as required by the Belgian law for involuntary commitment) with a manic episode of a bipolar I disorder; the hospitals that perform such an expert psychiatric assessment in the Brussels region of Belgium use the criteria in the DSM‐IV as a reference for reporting the diagnosis; the patient undergoes the psychiatric expertise in one hospital and is then transferred to another hospital for the involuntary commitment, which lasts for 40 days on average. As a routine, blood and urine analysis were realized as part of the early clinical evaluation of patients to exclude a manic symptomatology secondary to another problem (e.g., drugs). All included patients were not taking any treatment on admission, either because they stopped treatment themselves before admission, or because they never had treatment before: only 6% of patients were committed for the first time, while the rest (94%) of patients were committed before.

All manic patients included in the study were examined and followed by the same psychiatrist (the first author of this study) and were treated with a standard set of drugs following the local protocol for the treatment of manic patients: In the first stage of treatment (when the clinical presentation is severe), an association of typical and atypical antipsychotic drugs is administered, with a mood stabilizer as well as soporific and anxiolytic drugs when necessary and depending on the symptoms presented. In the second stage of treatment and when the clinical presentation is stable, patients are usually left with an atypical antipsychotic, a mood stabilizer, as well as a soporific and/or anxiolytic drug when necessary. The data sets supporting the findings of this study are available from the corresponding author upon reasonable request.

#### 2.1.2 Measurement

The YMRS (Young et al., 1978) is used in our ward to assess the severity of mania as a part of the routine clinical examination and report. Three time points for each item of the YMRS were collected for each patient, each time points: on admission (*t*_0_), halfway through the commitment period (*t*_1_, usually on day 20), and on discharge (*t*_2_, usually on day 40). The symptoms assessed (Table 1) are “Elevated Mood,” “Increased Motor Activity‐Energy,” “Sexual Interest,” “Sleep,” “Irritability,” “Speech (Rate and Amount),” “Language‐Thought Disorder,” “Content,” “Aggressive Behavior,” “Appearance,” and “Insight.” Symptoms were scored 0–4, depending on the severity of the clinical presentation, both at *t*_0_, *t*_1,_ and *t*_2_. Although some symptoms in the YMRS are scored from 0 to 8, all variables were scored from 0 to 4 when inputting the data because we were only interested in estimating a variance–covariance matrix as an input for the network model, and the YMRS sum score itself was not a variable of interest in this study.

**Table 1:** Descriptive statistics for the eleven symptoms in the three time points

	Mood0	Motor0	Sexual0	Sleep0	Irritable0	Speech0	LgTtAbn0	Content0	Aggressive0	Appearance0	Insight0
Mean	3.630	2.870	0.810	2.650	2.560	2.860	2.330	2.580	2.090	1.620	3.190
Std. Deviation	0.787	1.203	1.440	1.218	1.282	1.198	1.264	1.304	1.712	1.556	1.412
Minimum	0.000	0.000	0.000	0.000	0.000	0.000	0.000	0.000	0.000	0.000	0.000
Maximum	4.000	4.000	4.000	4.000	4.000	4.000	4.000	4.000	4.000	4.000	4.000

	Mood1	Motor1	Sexual1	Sleep1	Irritable1	Speech1	LgTtAbn1	Content1	Aggressive1	Appearance1	Insight1
Mean	2.360	1.720	0.470	1.860	1.640	1.750	1.260	1.540	1.130	0.910	2.530
Std. Deviation	0.644	0.766	0.870	0.853	0.772	0.702	0.799	0.809	0.884	0.889	1.185
Minimum	0.000	0.000	0.000	0.000	0.000	0.000	0.000	0.000	0.000	0.000	0.000
Maximum	3.000	3.000	3.000	3.000	3.000	3.000	3.000	3.000	3.000	3.000	4.000

	Mood2	Motor2	Sexual2	Sleep2	Irritable2	Speech2	LgTtAbn2	Content2	Aggressive2	Appearance2	Insight2
Mean	1.110	0.560	0.140	1.050	0.430	0.520	0.320	0.420	0.120	0.220	1.600
Std. Deviation	0.737	0.625	0.427	0.757	0.573	0.627	0.548	0.622	0.409	0.524	1.356
Minimum	0.000	0.000	0.000	0.000	0.000	0.000	0.000	0.000	0.000	0.000	0.000
Maximum	2.000	2.000	3.000	3.000	2.000	2.000	2.000	2.000	2.000	2.000	4.000

### 2.2 Network analysis

#### 2.2.1 Software

The software used for the analyses carried out in this study is R (version 3.6.3, available at https://r‐project.org). The packages needed for the analyses were bootnet (Epskamp et al., 2017), qgraph (Epskamp et al., 2012) for network estimation, visualization and stability, psychonetrics (Epskamp, 2019) for temporal network estimation, mgm (Haslbeck & Waldorp, 2016) for network inference, and Network Comparison Test (van Borkulo et al., 2016) for comparing network structures at different measurement occasions.

#### 2.2.2 Network estimation

##### Cross‐sectional networks

A cross‐sectional network model, or Gaussian graphical model (GGM), is a partial correlation network and is estimated as the inverse of the variance–covariance matrix. Three separate GGMs were estimated for *t*_0_, *t*_1_, and *t*_2_ data sets. In each GGM, each node representing a symptom in a network is connected through an edge with all other nodes: The strength of the connection, or edge weight, is determined by the partial correlation that is shared between the two variables (Epskamp et al., 2017). Each edge weight can be positive (denoting a positive association between two variables) or negative (denoting a negative association between two variables). The edges in a GGM are undirected, that is, we do not know if a symptom causes or is caused by other symptoms sharing a connection with it. The presence of an edge in a network structure is interpreted as the existence of a conditional dependence between two variables: If A and B are connected, they influence each other given all other nodes in the network. Although most network structures in the literature are estimated using a regularization procedure (e.g., the lasso), we did not use regularization in our study following recent recommendation that applies to psychiatric data (Williams & Rast, 2019).

##### Temporal network

A panel graphical vector autoregressive model (GVAR) recently introduced in the network literature (Epskamp, 2019) was used to model the dynamics of manic symptoms with a pharmacological intervention in three time points *t*_0_, *t*_1_, and *t*_2_. GVAR can be seen as a multivariate multiple regression on the previous measurement occasion; a nonzero element in the temporal matrix means that a given variable B is predicted over time by another variable A: this prediction is known as Granger causality (Granger, 1969), because the condition of “cause preceding the effect” is fulfilled. Temporal effects can also be positive or negative.

#### 2.2.3 Network inference

Node predictability was estimated for the 11 symptoms in the three data sets and is reported in Table 2. Node predictability represents shared variance of a given node with surrounding nodes in a network, that is, what percentage of variance of a given node can be explained by other nodes (Haslbeck & Fried, 2017). It can be interpreted as an absolute measure of how well a node is connected in the network (Briganti et al., 2019).

**Table 2:** Node predictability estimates for the eleven symptoms at the three time points

Motor	0.374	0.138	0.168
Sexual	0.000	0.000	0.000
Sleep	0.312	0.177	0.077
Irritable	0.352	0.240	0.215
Speech	0.173	0.120	0.252
LgTtAbn	0.291	0.278	0.485
Content	0.224	0.220	0.443
Aggressive	0.307	0.268	0.164
Appearance	0.201	0.177	0.000
Insight	0.236	0.211	0.228

The Network Comparison Test (NCT) was performed to compare global strength in the three networks (van Borkulo et al., 2016). Three tests were performed, respectively, to compare *t*_0_ and *t*_1_, *t*_1_ and *t*_2_, and *t*_0_ and *t*_2_.

Stability and accuracy analyses for the cross‐sectional network structures were carried out following guidelines in network analysis (Epskamp et al., 2017).

### 2.3 Ethical approval

The protocol for this study was approved from the Ethical Committee of the Brugmann Teaching Hospital in Brussels (CHU Bruxelles Brugmann), with the reference number CE 2020/39 and the project title “Modeling severe mental disorders using complex systems and machine learning.”

## 3 RESULTS

### 3.1 Descriptive statistics

Patients were 20–72 years old (*M* = 44.5, *SD*=:14.5); 47% of them were female, and 53 of them were male. The descriptive statistics for each symptom at each time point are reported in Table 1.

### 3.2 Cross‐sectional networks

**Figure 1:**
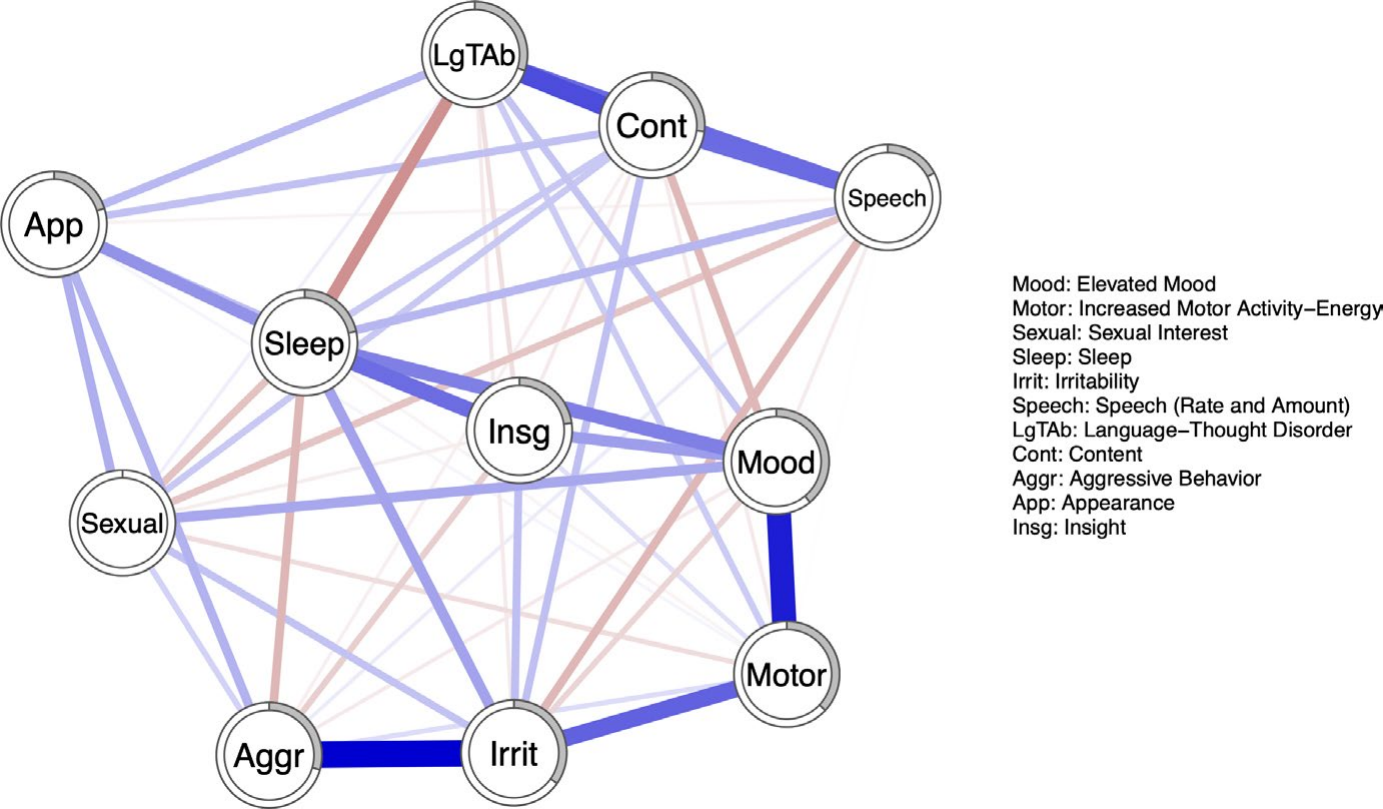
Network structure of manic symptoms at *t*_0_. Each node represents one of the eleven items from the YMRS. Blue connections represent positive edges, and red connections represent negative edges. The pie chart surrounding each node represents node predictability

**Figure 2:**
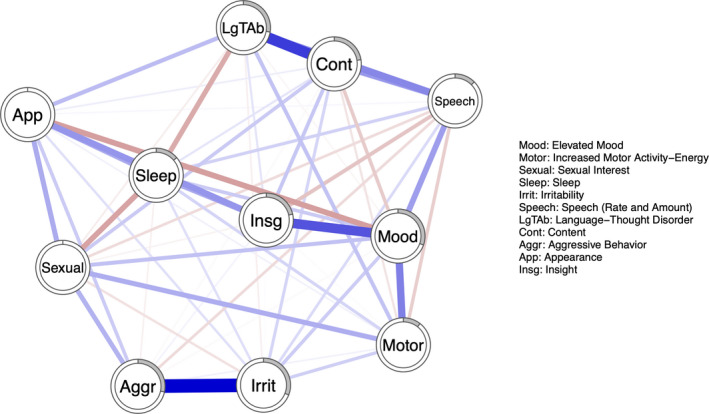
Network structure of manic symptoms at *t*_1_. Each node represents one of the eleven items from the YMRS. Blue connections represent positive edges, and red connections represent negative edges. The pie chart surrounding each node represents node predictability

**Figure 3:**
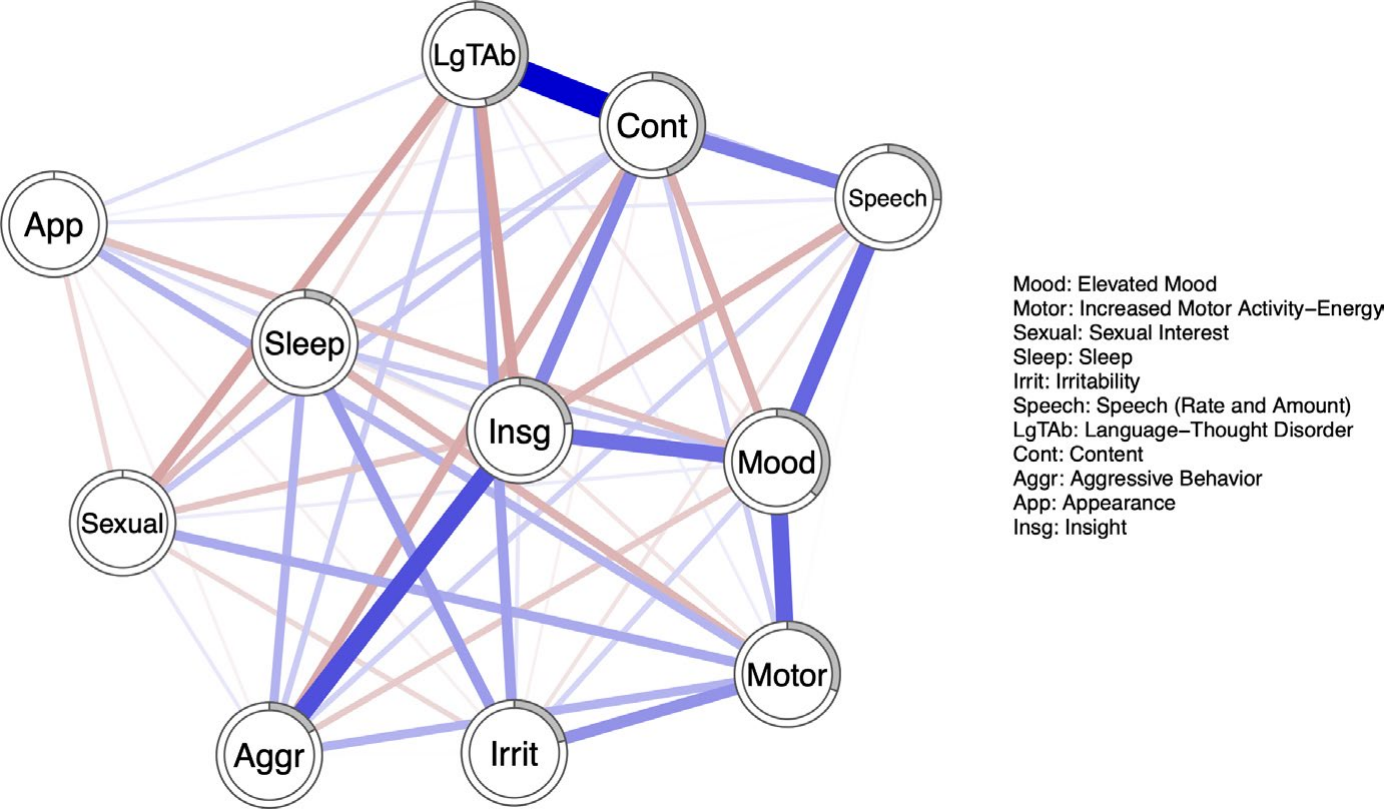
Network structure of manic symptoms at *t*_2_. Each node represents one of the eleven items from the YMRS. Blue connections represent positive edges, red connections represent negative edges. The pie chart surrounding each node represents node predictability

**Figure 4:**
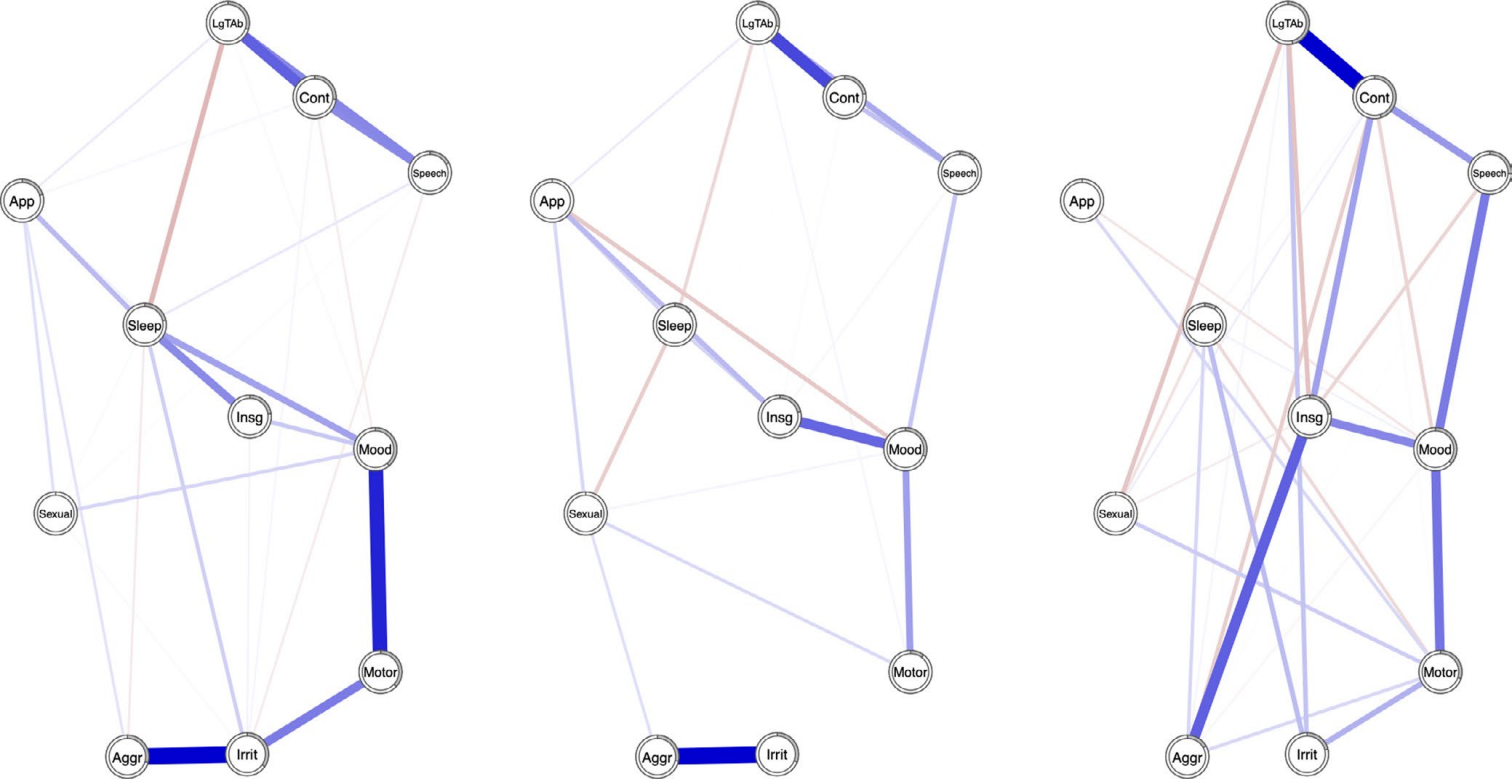
Network structure of manic symptoms at *t*_0_, *t*_1_, and *t*_2_. Each node represents one of the eleven items from the YMRS. Blue connections represent positive edges, and red connections represent negative edges. The pie chart surrounding each node represents node predictability. Only edges with a weight greater than 0.1 are reported

The three network structures of manic symptoms at *t*_0_, *t*_1_, and *t*_2_ are illustrated in Figures 1, 2, 3. Figure 4 represents the three networks side by side. Overall, the networks present both positive and negative connections; however, the relative strength of connections (compared to other connections in the same network) in the respective networks varies over time. Hereby, some of the connections are described.

Aggressive behavior shares a strong connection to irritability at *t*_0_ and *t*_1_; however, at *t*_2_, it shares a connection with Insight instead. Language‐thought disorder is highly connected to content at all time points; content is also connected with speech at all time points. Elevated mood shares connections with motor activity and insight.

### 3.3 Network inference

**Figure 5:**
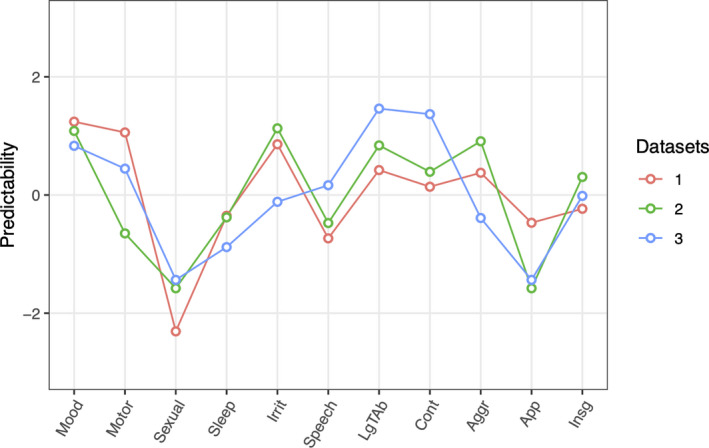
Network predictability values for the *t*_0_ (red line), *t*_1_ (green line), and *t*_2_ (blue line) networks a standardized z values

Network predictability estimates for the three time points are reported in 3 and represented in Figure 5. Elevated mood is the most interconnected node at *t*_0_ (Increased Motor Activity – Energy is a close second) and language‐thought disorder is the most interconnected node at *t*_1_ and *t*_2_.

The NCT reported a statistically significant difference between the global strength of *t*_0_ and *t*_2_ (*p* = .0014); however, *t*_0_ and *t*_1_, as well as *t*_1_ and *t*_2_ do not present significant differences in global strength (*p* = .58 and *p* = .98, respectively).

### 3.4 Temporal network

**Figure 6:**
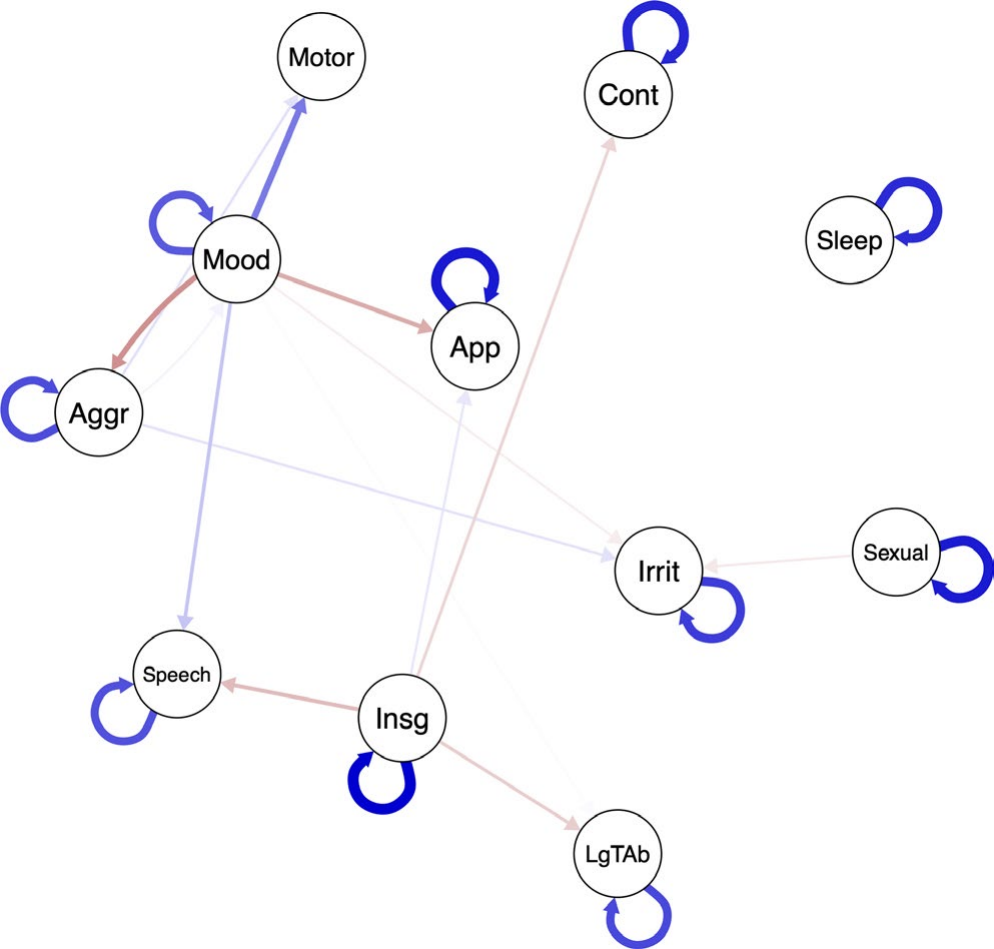
Temporal network

The temporal network estimated through the GVAR adapted for panel data is represented in Figure 6. However, there are several variables in the network that are Granger‐caused by many other variables over time, three examples of such variables are sleep, mood, and increased motor activity. Other variables, such as insight, receive few temporal effects.

## 4 DISCUSSION

This work tackled the important issue of the study of mania as a network of symptoms while using the YMRS to assess manic symptoms.

The strong connection between aggressive behavior and irritability at the two time points *t*_0_ and *t*_1_ means that, when mania is untreated, the two symptoms are highly predictive of one another; if patients are irritable, then there will be a high chance for them to be aggressive as well, and vice versa. However, at *t*_1,_ aggressive behavior becomes predictive of insight instead; this can be interpreted as a hint that stable manic patients that tend to have no insight, also tend to show greater signs of aggressive behavior (even though the mean value for aggressive behavior at *t*_2_ is close to 0 in our sample), controlling for all other symptoms in the network.

Language‐thought disorder and content are highly connected at all time points; from a network point of view, this means that the presence of an abnormal communication with the patient (due to, for instance, flight of ideas or echolalia) may suggest that there is an ongoing delusional or hallucinatory process (and vice versa), controlling for all other symptoms. The association of language‐thought disorder and delusions or hallucinations is recurrent in the literature; recent works on psychosis also suggest that the less patients are in control of their thought process, the more likely they suffer from hallucinations (Hartley et al., 2015). Another possible red flag linked to delusions or hallucinatory processes may be found in speech alterations, such as logorrhea, because of the association between speech and content at all time points.

Elevated mood is connected with an increased motor activity and insight; although this is not very informative during severe manic episode which is the case at *t*_0_ (because an elevated mood is the core feature of mania), it informs the clinician that when the patient is stable (*t*_2_) a loss of insight (if not present before) or an increased energy may be the sign of a rising mood; this finding is supported by recent literature (Silva et al., 2018).

At *t*_0_ elevated mood is the most interconnected symptom in the manic network. Because edges are not directed in the partial correlation networks, elevated mood can be interpreted as the symptom that best predicts (or is predicted by) all other symptoms in the network at *t*_0_. However, when patients become more stable, language‐thought disorder becomes the most predictable node in the network; this is likely due to the strong connection that it shares with content, which also presents high estimates at *t*_1_ and *t*_2_. This phenomenon is described as “centrality corruption” (Briganti & Linkowski, 2020); that is, because two nodes share one strong network connection, they rapidly become important in the self‐determination of the network. For this reason and because it shows a high estimate when patients have a severe mental state, the connectivity of elevated mood at *t*_0_ is more straightforward to interpret, as it shares connections with several nodes; elevated mood can be considered as the core feature of mania in our sample of manic patients.

Psychomotor agitation (“Increased Motor Activity – Energy”) is also highly connected at *t*_0_: this mirrors previous findings in the literature (Weintraub et al., 2020); however, in our sample, elevated mood is the most interconnected symptom at *t*_0_; this may be explained by our sample population of severe manic patients which therefore present a severe mood alteration.

The NCT reported a statistically significant difference between the global strength of networks at *t*_0_ and *t*_2_. This means that the network presents a different connectivity at the two time points; it is worthy of note that the networks compared by NCT are slightly different than the ones estimated, because they have regularized partial correlation estimated with a Pearson's rho input. Although other comparisons between network structures are described in this work, the results from NCT offer supplementary arguments in favor of a difference in network structures on admittance and discharge.

Granger causal effects were explored in the temporal network. Three examples of variables that receive many effects are sleep, increased motor activity and mood. From a network point of view, it is interesting to see how other variables have temporal effects on mood, which from a categorical perspective, is supposed to be the direct representation of the cause, the mood disorder itself; mood receiving many incoming edges in the temporal network supports a complex system view of the concept of mania.

The results of this work should be interpreted in light of five limitations. First, the assumption of stationarity (the variables have the same mean and the same standard deviation over time) is likely violated in our sample, because patients present high scores at *t*_0_ and low scores at *t*_2_: this leads to a less good model fit. Second, and for the same reason regarding patient scores, Spearman's *ρ* correlation was used instead of Pearson's as an input for the GGM because the distribution of the data is skewed. Third, although GVAR can be estimated from three measurement occasions, it would be optimal to obtain a sample with many more time points, and with more subjects to obtain more accurate estimates. Fourth, all patients included in this study were examined by one physician, which is a potential limitation for the reliability of the measures obtained from the routine YMRS interviews. Five, the nature of our patient sample is extremely rare, which is likely to limit the generalizability of our findings.

## 5 CONCLUSIONS

This study expanded the network theory of mental disorders to mania. Manic symptoms were interconnected in a network structure, and specific associations between symptoms, both static (cross‐sectional) and dynamic (temporal) nature were investigated, as well as the importance of symptoms in the self‐determination of the network. Future work may endeavor to replicate our results in other population, as well as in patient with a less severe condition.

## CONFLICT OF INTERESTS

None.

## AUTHOR CONTRIBUTIONS

Giovanni Briganti and Charles Kornreich collected the data. Giovanni Briganti performed the statistical analyses and drafted the manuscript. Paul Linkowski and Charles Kornreich reviewed the manuscript.

### Peer Review

The peer review history for this article is available at https://publons.com/publon/10.1002/brb3.2010.

## Data Availability

The data that support the findings of this study are available from the corresponding author upon reasonable request.
